# Procalcitonin as a predictive marker for PCR test and blood culture results in suspected invasive candidemia

**DOI:** 10.1186/cc10636

**Published:** 2012-03-20

**Authors:** A Cortegiani, SM Raineri, F Montalto, MT Strano, A Giarratano

**Affiliations:** 1 University Hospital Policlinico P. Giaccone, Palermo, Italy

## Introduction

Procalcitonin (PTC) seems to have potential to predict the result of blood culture (BC) supporting the diagnosis of invasive candidemia. Although blood culture is still the gold standard, PCR assays are able to quickly and reliably detect fungi in blood in suspected invasive candidemia. Our aim is to verify the potential of PTC values to predict the result of PCR assay in suspected invasive candidemia.

## Methods

We retrospectively analyzed 78 patients with suspected invasive candidemia from whom we obtained PCT value, BC and PCR assay. All tests have been obtained on the day in which patients reached a Candida score ≥4. We calculated PTC mean values according to BC and PCR results and compared data using the Mann-Whitney U test. We performed the ROC analysis to test the diagnostic performance of PTC with regards to BC and PCR result.

## Results

PCR tests and BC were both negative in 48 patients and the PTC mean value in this group was 21.5 ng/ml while 19 patients were PCR-positive and BC-positive with a PTC mean value of 2.07 ng/ml. The difference between these PCT mean values was significant (*P *= 0.0001). In eight cases BC were negative whereas PCR tests were positive with the PCT mean level in this group being 1.82 ng/ml. No patient resulted PCR-negative and BC-positive. According to PCR results only, there was a significant difference between PTC mean values in positives and negatives (*P *= 0.0001). The ROC analysis showed that the best PTC cut-off value for prediction of BC result was 4.57 with AUC of 0.91 (CI 0.83 to 0.96, sensitivity 99%, specificity 80.39%). Concerning the PCR result, the calculated cut-off was 4.31 with AUC of 0.96 (CI 0.948 to 1, sensitivity 96.6%, specificity 97.9%; positive predictive value 94.51%; negative predictive value 97.83%).

## Conclusion

According to our data, PTC seems to be characterized by a remarkable diagnostic performance and predictive value for both BC and PCR assay in suspected invasive candidemia. PCT could be considered as the first step of the diagnostic process for suspected invasive candidemia in order to spare as much time as possible before starting a pre-emptive antifungal therapy. This may lead to less useless therapies in negative patients and quicker and more reliable start of treatment in positive patients while waiting for the BC and antibiogram results.
